# Inclusive approaches to involvement of community groups in health research: the co-produced CHICO guidance

**DOI:** 10.1186/s40900-023-00492-9

**Published:** 2023-09-07

**Authors:** Catherine Jameson, Zehra Haq, Samira Musse, Zahra Kosar, Gloria Watson, Vikki Wylde

**Affiliations:** 1 Musculoskeletal Research Unit, Bristol Medical School, Southmead Hospital, University of Bristol, Learning and Research Building, Bristol, BS10 5NB UK; 2 NIHR Bristol Biomedical Research Centre, University Hospitals Bristol and Weston NHS Foundation Trust and University of Bristol, Bristol, UK; 3 Dhek Bhal, Wellspring Settlement, Barton Hill, Bristol, UK; 4 My Friday Coffee Morning – Barton Hill, University of Bristol micro campus, Barton Hill, Bristol, UK; 5 Malcolm X Elders, Malcolm X Community Centre, St Pauls, Bristol, UK

**Keywords:** Ethnic groups, Community groups, Diversity, Involvement, Health research, Guidance

## Abstract

**Background:**

Racially marginalised groups are underserved in healthcare and underrepresented in health research. Patient and public involvement and engagement (PPIE) is established as the method to ensure equity in health research. However, methods traditionally employed in PPIE can lead to the exclusion of some communities and exacerbation of existing inequalities, highlighting the need to develop inclusive processes for more inclusive community involvement in health research. We aimed to produce guidance to promote good practice for inclusive involvement of racially marginalised community groups in health research via public and community involvement and engagement.

**Methods:**

The CHecklist for Inclusive COmmunity involvement in health research (CHICO) was co-produced by researchers and three Bristol-based community organisations: Dhek Bhal, My Friday Coffee Morning—Barton Hill, and Malcolm X Elders. After initial conversations and link building with community leaders to develop relationships, researchers attended at least three meetings with each community group to discuss preferred approaches to involvement. Each community group had a different format, and discussions were open and tailored to fit the groups preferences. The meetings were held in the community groups’ usual meeting venue. Notes from meetings were reviewed by researchers to identify key themes, which were used to inform the creation of a draft illustration which was then taken back to the community groups for refinement and used to inform the development of written guidance and the final illustration.

**Results:**

Checklist items were structured into three stages: (1) building relationships, (2) reciprocal relationships and (3) practicalities. Stage 1 highlights the importance of building trust with the community group over time through regular visits to community venues and talking to people informally to understand the history of the group, their preferences and needs, and topics that are likely to be of interest to them. Stage 2 focusses on maintaining a reciprocal relationship and understanding how to best to give back to the community. Stage 3 provides guidance on the practicalities of designing and running inclusive community-based involvement activities, including consideration of the venue, format, communication-style, language requirements, social activities, and provision of food.

**Conclusions:**

Our co-produced checklist can guide researchers in how to involve people from different ethnicities in health research that is relevant to their community.

**Supplementary Information:**

The online version contains supplementary material available at 10.1186/s40900-023-00492-9.

## Abstract (plain language summary)

Health research needs to be for everyone. For this to happen, people from all backgrounds need to be involved. However, people from ethnic minority backgrounds are often not involved in the design of health research. We wanted to find out how researchers and community groups can work together to make the research process more inclusive. Researchers worked with three community groups in Bristol. These were South Asian, African Caribbean and (majority) Somali community groups. One researcher talked with community leaders to find out if the interests of the research unit was relevant to community members and to understand how each group would prefer to work. Following this, at least three meetings were held with each group. After these meetings, we looked at what was talked about. We suggest that researchers involving community groups in health research follow three key steps. Step 1 is making relationships and covers the importance of building trust with the community. This should be through talking with community leaders and regular visits to the group to get to know members before asking for input. Step 2 is about how to best to give back to the community. This is important to make sure that relationships are fair. Step 3 is about how to design and run activities. This includes thinking about the venue, language needs, social activities, and providing food. Our suggestions can help researchers and community groups to work together on health research. We have made an illustration of our findings for sharing.

**Supplementary Information:**

The online version contains supplementary material available at 10.1186/s40900-023-00492-9.

## Background

In the UK, people from ethnic minority backgrounds have traditionally been marginalised and therefore underserved in healthcare. For example, within elective orthopaedics, there are widespread inequalities in healthcare, with people from ethnic minority groups less likely to receive joint replacement than people who are white [1–3]. There are also ethnic disparities in outcomes after joint replacement, with patients from ethnic minority groups experiencing greater post-operative pain and disability, higher rates of complication and increased mortality [2–4]. These inequalities are likely to be exacerbated following the COVID-19 pandemic, which disproportionately affected people from ethnic minority groups, who were more likely to have heightened exposure during the pandemic and live with conditions associated with an increased risk of illness from COVID-19 [5].

It is now widely acknowledged that study sample populations in health research need to reflect the communities that they serve to ensure equity and to fully understand differences in treatment responses, cultural context, and relevance [6]. However, people from ethnic minority groups continue to be underrepresented in health research, despite comprising a large proportion of the UK population [6–8]. The most commonly identified strategy for improving the participation of underserved groups in health research is through the involvement of patients, public and communities in the research process [9]. Involvement is defined as an activity that is done ‘with’ or ‘by’ patients or members of the public rather than ‘to’, ‘about’ or ‘for’ them [10]. Commonly known as Patient and Public Involvement and Engagement (PPIE), a more inclusive term which reflects the diversity of involvement is Patient, Public and Community Involvement and Engagement (PCIE) [9]. A report by the National Institute for Health Research (NIHR) highlighted that inclusion in PCIE needs to be addressed, otherwise there is a significant risk that health inequalities and discrimination will worsen [11]. Developing inclusive processes for PCIE is fundamental to designing and delivering health research that meets the need of all who could benefit and addresses health inequity [12].

Methods traditionally employed in PPIE can lead to the exclusion of some communities and exacerbate existing inequalities [13, 14]. Structural racism, which involves structures which exclude members of some social groups from full participation in society, imposes complex barriers to involvement. Examples include researchers’ unconscious bias, power dynamics, mistrust, use of academic language and jargon, assumed high levels of literacy in English, rigid agendas and formal presentation styles, requirement to pre-read materials, holding meetings online, travel to in-person meetings, meetings in work hours, and lack of communication support e.g. interpretation or translation [14–17]. Understanding how PCIE can be done in a way that is accessible and inclusive to racially marginalised communities is an important contributing factor to addressing health inequalities. We aimed to develop practical and accessible guidance for researchers to promote good practice for inclusive involvement of communities in health research.

## Methods

Reporting of PCIE activities in this manuscript follows guidance from GRIPP2 [18], which describes the key items to report to enhance the quality, transparency, and consistency of PCIE in health research. A completed GRIPP2 short-form is provided in Additional file 1: Appendix 1. This article reports on PCIE activities to co-develop a checklist and therefore institutional ethics approval was not required.

The checklist was co-produced by two academic researchers (Catherine Jameson and Vikki Wylde) and three Bristol-based community organisations: Dhek Bhal, My Friday Coffee Morning—Barton Hill, and Malcolm X Elders. Catherine Jameson, a white British woman, is a Senior Research Associate in PCIE, with 21 years’ experience in PCIE and health research. Vikki Wylde, a white British woman, is a Professor of Musculoskeletal Health, with 19 years’ experience of working in musculoskeletal research. Dhek Bhal is a charity providing respite breaks, day care, self-help and advocacy activities for the South Asian community in Bristol and South Gloucestershire (https://www.dhekbhal.org.uk/). As part of the work of Dhek Bhal, weekly group meetings are held for older members of the local South Asian community to provide an opportunity for people to come together in a social space. The meetings are held at the Wellspring Settlement at Barton Hill in Bristol, with men and women meeting separately, and the Chief Executive is co-author Zehra Haq. The number of community members attending the weekly Dhek Bhal meetings varies but there was approximately 5–10 members that attended the men’s group and between 15 and 20 that attended the women’s group during this project. My Friday Coffee Morning—Barton Hill is a weekly drop-in coffee morning for the local community run at the University of Bristol microcampus as part of the Wellspring Settlement in Barton Hill, Bristol. The coffee mornings are facilitated by two members of the local community (co-authors Zahra Kosar and Samira Musse), and the coffee mornings are attended by approximately 10–15 local women of all, but majority Somali, heritages. Malcolm X Elders describe themselves as a weekly drop-in community group for African Caribbean elders. Attended by approximately 30 people, the weekly meetings are held in Malcolm X Community Centre in St Pauls, Bristol, and the organiser is co-author Gloria Watson.

Catherine Jameson and Vikki Wylde received funding from the Elizabeth Blackwell Institute at the University of Bristol to conduct a parcel of work to build meaningful relationships with local community groups and understand how we can best involve people from racially marginalised communities in health research. Community leaders are the gateway and advocates for their community groups, and therefore the first step was to identify and be introduced to community leaders. Initial conversations with community leaders were held to build links, develop relationships and plan the format and reward for the meetings, which involved a combination of face-to-face discussions in the community, telephone calls and email. Following these initial discussions, Catherine Jameson attended at least three meetings with each community groups to discuss preferred approaches to involvement activities. Each community group had a different format, and discussions were open and tailored to fit with the preferences of the group. An introduction was given by Catherine Jameson on the work of the Musculoskeletal Research Unit and her role. This was followed by an explanation that we knew that researchers had been to the group(s) before, but that we wanted to go back to the beginning to ask that, if we wanted to involve the group members in guiding our research, could they tell us how we should best do this. The discussions were kept deliberately open to encourage sharing of views, confidence and trust building and so were often wide-ranging. Discussions that were off topic were acknowledged, answered where possible and noted for future discussion or research ideas. The meetings were held in the community groups usual meeting venue. Notes were taken by a researcher during each community group meeting. Members of the community groups were informed that notes were being made and that the purpose of the notes was to ensure that there was a written record of the discussions. The meetings were not audio-recorded and transcribed as it was important to ensure that the meetings were conducted in a way that was acceptable to community members so that they felt comfortable to openly share their thoughts and views with the researchers.

Meeting notes were reviewed by the two academic co-authors and a descriptive analysis undertaken to identify key themes that emerged from the community group discussions. The themes were tabulated, and group responses were compared. With the exception of translation and separate gender groups (not required for Malcolm X), common themes were raised by each of the community groups. Therefore, the decision was taken to produce one checklist from this project. The checklist was created from the themes that arose from the group discussions and checked and agreed by the community co-authors.

**Fig. 1:**
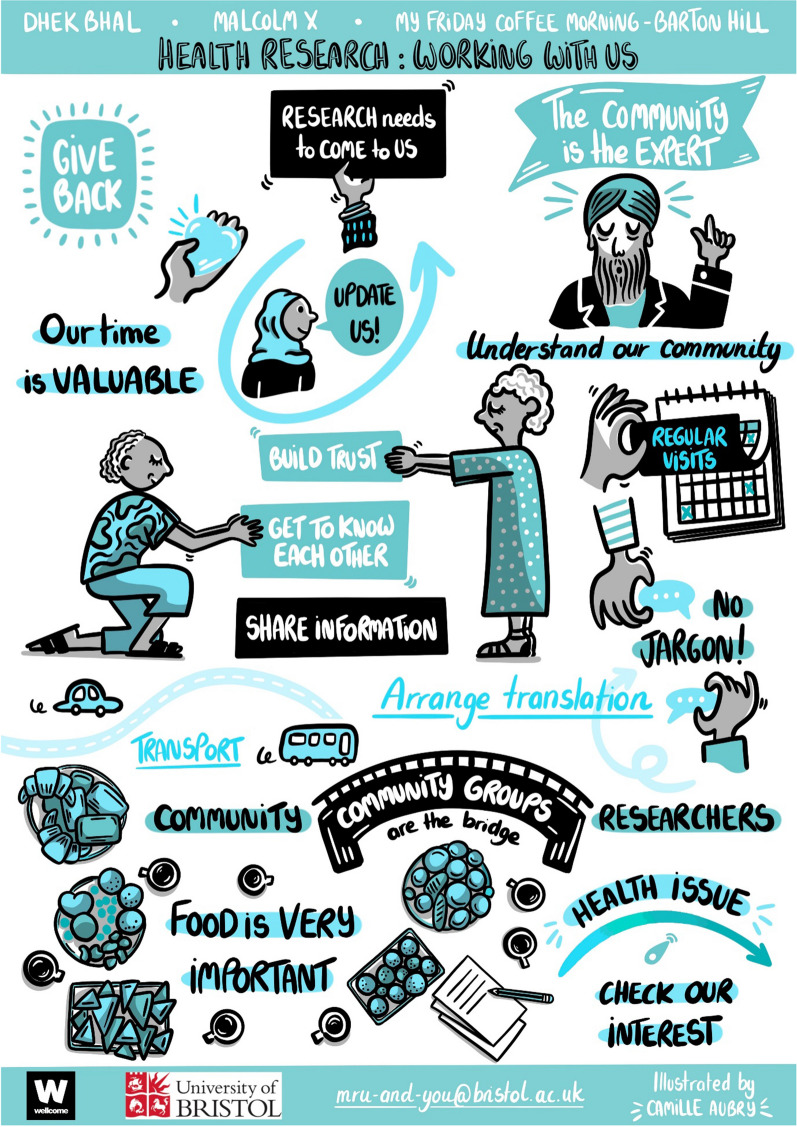
Illustration

A live illustrator attended some of the community group meetings and was also provided with notes, photographs and discussion to aid in the production of an illustration (Fig. 1). The original purpose of the illustration was to develop a resource that community groups could share with researchers who wanted to work with them, however it was also found to be a useful tool to engage the community groups in the analysis and interpretation of this work. The draft illustration, which provides a visual representation of the key themes, was shared and discussed with each community group, to inform refinements to the final checklist and illustration. Examples of refinements to the illustration included changing ‘relevance of topic’ to ‘health issue’ to improve plain language and changing ‘meetings’ to ‘visits’ as meetings was considered as too formal. The final illustration provides an output that can be shared with communities and other audiences.

## Results

The CHecklist for Inclusive COmmunity involvement in health research (CHICO), written in accessible language, is provided in Table 1, and the illustration is provided in Fig. 1. A GRIPP2 checklist is provided in Additional file 1: Appendix 1. Checklist items are grouped into three stages: 1. building relationships, 2. reciprocal relationships and 3. practicalities.

**Table 1:** CHICO: The CHecklist for Inclusive COmmunity involvement in health research

What the groups say	What this means for researchers
*Step 1: relationships*	
Group interest	Work with community leaders to gauge interest/relevance of topics before meeting
Understand our community	Work with community leaders to understand the needs of the group before meeting
Work with us in a way that suits us	Be flexible and informal and follow the lead of the community leader / group on format Bear in mind that a format that works for one group may not work for another
Build trust and relationships / get to know each other	Is there a key person who can work with the group? Take time to build relationships
Come back and see us	Have you factored in regular meetings to maintain the relationship with the group? Have you considered additional visits when you have no need for input?
Keep us in the loop	Have you planned to update the group on progress and outcomes of research? Show the group how they’ve made a difference
*Step 2: reciprocal relationship*	
Say thank you/our time is valuable	How can you say thank you? Agree with community leaders the best way to give back to the group
Give us the information we need/want	Have you considered ways to give added value? For example, organise visits from doctors/experts to discuss health issues important to the group
We are the experts	Listen to and acknowledge expertise—they are the experts in their own experience of healthcare provision
*Step 3: practicalities*	
Come to us	Are you meeting with the community groups at their usual time, day and location?
Use our language	Where applicable, have you asked groups which language(s) they would like the meeting to be conducted in, and how they would be most comfortable doing the translation? Pay for community leader(s)/external translator
Help us get there	Have you considered transport needs and pay for means for people to the attend group?
‘Food is very important’	Are you providing/paying for culturally appropriate food Consider using existing in-house or local providers
‘No fancy words’	Are you using plain language? Be aware of jargon—use real life examples
Let us enjoy our time together	Have you built in time for social time/activities? Ask the community leaders/group what they would like

### Stage 1: building relationships

Stage 1 highlights the importance of building trust with the community. In contrast to the now out of favour ‘hard to reach’ description for racially marginalised groups, these communities are frequently contacted by researchers asking for input. However, researchers often use a ‘parachute’ approach where they attend only to ask for input from the group with no focus on reciprocal and sustained relationships. This has led to some community members feeling used and therefore unwilling to work with researchers going forwards. This was shown by some community members being unwilling to talk to researcher Catherine Jameson on first visits, voicing that they did not believe that she would return. This meant that, for some community members in particular, repeat visits and conversations were needed before they felt comfortable speaking with the researcher, after which they stated that they would be happy talking with the researcher going forwards. During discussions, people voiced that the reason they want to get involved is to make a difference, often to make things better for other people as well as themselves. However, people also talked about how they felt that nothing changed as a result of them having previously being involved in research. These two issues can lead to people becoming disenfranchised and therefore further under-represented within research studies. Having the time to build relationships through regular visits to community venues to talk to people informally is essential to build back the trust that has been lost. This stage can take time and involves developing a relationship with the community leader(s) and the group before any requests for formal involvement activities. We found that initial conversations between the researcher and the community leader(s) helped to understand the history of the group, their preferences and needs, and topics that were likely to be of interest to them. Regular contact and visits to the group by the researcher is essential to get to know members and to maintain ongoing communication.. Visits with no set agenda were beneficial to get to know members and joining in with their usual activities helped with building trust. Allowing open discussion during visits and acknowledgement of their views was important to allow group members to feel comfortable sharing within the group situation and/or with the researcher. Eating food together is a powerful leveller and allows for further social interaction; we found that eating together took the focus away from work and allowed for more personal conversation, thus strengthening the relationships.

Change in NHS practices and care as a result of research studies can take years, so returning to feedback on the research studies that they have had input into shows people that they are making a difference. Visits need to be ongoing to build and maintain long-term and meaningful relationships. Through repeat visits to each community group, both to spend time with the groups for non-academic events and social events, as well as to discuss research, we have fostered ongoing relationships built on trust and reciprocity.

### Stage 2: reciprocal relationships

Stage 2 should be an ongoing and iterative process to understand how best to build and maintain reciprocal relationships and give back to the group. Members of the community groups shared with us that rather than never being asked for input by researchers, they experience a constant stream of requests. However, researchers often never returned to share what difference their involvement had made, resulting in one-sided relationships that were only beneficial to the researchers and research institutions. Relationships with community groups should be a two-way relationship, with efforts made to understand how best to acknowledge the value of people’s input and thank them for their contribution. This may be financial, for example paying individual group members for their time, paying for lunch, making a payment to the community group and/or funding social activities. The usual or ‘transactional’ model of PPI is that each individual is paid for their time, and whilst we support this, the researchers discussed with community leaders on how to give back on this consultation. For Dhek Bhal and Malcolm X Elders, it was decided that the community group would benefit most from payment towards catering, subsidising or paying for activities and transport rather than individual vouchers. With sometimes up to 40 members attending Malcom X Elders, individual payment would also have made the costs prohibitive. The format of the My Friday Coffee morning was already set up as a group for the women to come together and discuss important topics as well as socialising, with community leader payment, provision of university space and breakfast. The researchers have also used their networks to address a particular health or social need that the group has expressed, for example arranging for a physiotherapist to run an exercise session after group members expressed an interest in learning more about maintaining musculoskeletal health. Where community group members have subsequently become involved in guiding specific research projects, the ‘usual model’ of individual payment has been used. We recommend a combination of these ways to thank the groups for their time. Importantly, it is crucial to feed back to communities about the changes that have resulted from their valuable contribution and the impact this has had on research.

### Stage 3: practicalities

The items in Stage 3 provide guidance on the practicalities of developing and running inclusive community group involvement activities. A key element is ensuring that the location and format of activities is discussed with the community leader(s) in advance to understand what would work best for that particular community group, for example embedding activities within existing community meetings at their usual venue. Group members feel more comfortable attending their local community venue and holding discussions at existing meeting times mean that we avoid barriers such as transport, care responsibilities and language. Given that we knew that these groups were already marginalised and that the research community have put barriers in the way of community groups, we wanted to break from the ‘usual’ model of PPI, ie selecting people or allowing people with particular experiences to self-select via advertisement and inviting them to meet to discuss research at a university location or over virtual meetings. Travelling to meet with existing community groups meant that relationships were built with members of the communities that may not have attended usual model PPI meetings, eg those who don’t speak English, are frail, elderly, have caring responsibilities, don’t use a mobile phone/internet/are digitally excluded and/or who are already disenfranchised. Flexibility in approaches to working is important as we found that each community group had different preferences and different meeting formats. To avoid overburdening, it was important to factor in time for social activities, with protected time for usual group activities if the PCIE activities are being accommodated within an existing community group meeting. Provision and sharing of food is viewed as very important and should be provided at meetings. It was voiced by two groups (Dhek Bhal and Malcolm X Elders) that using existing providers or local providers ensured culturally appropriate provision and to kept resources within the local community. Clear communication during the meeting is essential; for example group members said ‘don’t use fancy words’. We therefore we recommend that research or medical jargon should be avoided, and efforts need to be made to bring in real life examples that the group will be able to relate to, for example in place of ‘musculoskeletal’ we said ‘bones and joints’. Language may need to be considered; this requires discussion with the community leader(s) about what language(s) the meeting will be held in, and preferences for translation. Be aware that within community groups there may be a number of languages spoken and within those, different dialects. If translation is needed, it is important to discuss who will provide this, for example the community leader(s), a member of the community group or an external translator, and researchers should arrange to pay for any associated costs. Dhek Bhal group meetings were held in the languages that were usually spoken and if translation was required, it was from the group into English, rather than assume that the meeting should be in English and translated to the language/s of the group. Researchers also need to consider transport, and how people can be supported to attend the meeting, being guided by the community leader(s) and factoring in appropriate budget.

## Discussion

Working as a partnership between researchers and community groups, we have developed CHICO to guide researchers on how to involve community groups meaningfully and inclusively in health research. Spanning over three key phases, the checklist provides guidance on how to build initial trust and relationships with community groups, how to conduct acceptable and inclusive involvement activities and then how to ensure and maintain a reciprocal relationship. Involvement of communities should not be approached on a project-by-project basis, instead long-term and sustained relationships are needed to rebuild trust and address a history of discrimination and inequities. The importance of individuals, such as a designated PCIE coordinator, having protected time to nurture and maintain these relationships is paramount to ensuring that the valuable contribution of racially marginalised communities can shape the nation’s health research.

The COVID-19 pandemic starkly highlighted the need to improve the involvement of underserved groups in health research, given that they were disproportionately affected by COVID-19 but underrepresented in research studies [20]. There is a clear need to address these inequities and ensure that people who are marginalised in health research are involved in all stages of the research process, from research prioritisation to dissemination [12]. There is a growing literature on widening inclusion in PCIE, with innovative methods, toolkits and guidance. Examples of innovative approaches include using creative methods to facilitate involvement of people with communication difficulties [13] and community sandpit events to build relationships with community organisations and fund community-led innovation [16]. Good practice guidelines have been developed to increase the participation of BAME (Black and Minority Ethnic) groups in health and social care research [21]. These include six recommendations, one of which is to undertake effective involvement activities. We build on this recommendation and provide practical guidance on how to build partnership with community organisations and conduct inclusive involvement activities to inform health research. Previous research has identified facilitators to involvement of ethnic minority communities in health research. In a systematic review on involvement of black and minority ethnic groups in health and social care research, Dawson and colleagues identify that allowing time for researchers and community members to build relationships and trust, understanding the individual needs and concerns of people, bilingual researchers, open agendas and allowing time to listen and discuss health issues, and providing compensation as recognition for people’s contribution were facilitators to involvement [12]. A recent systematic review of reviews identified similar facilitators and highlighted the importance of independent facilitation of involvement activities, and open and honest communication [17]. It is important to note that, for this project, we did not pay individual group members. Whilst we use this approach, as detailed by the NIHR[22], for other PPI work we carry out, it was decided by the group leaders that it was appropriate to support the whole group in other ways. These methods included paying the community group for facilitator/translator time, contribution towards group social activities and provision of food from in-house or local providers. This was noted as being important for maintaining the longevity of these valued community group. We also continue to bring added value to the groups via our ongoing relationships, for example, providing experts to answer their health questions and funding non-academic sessions where communities decide on a speaker to attend. This approach is also recommended as an option in the NIHR guidance. We recommend being flexible and guided by the groups and group leaders as to the best method for reward.

It is important to consider the strengths and limitations of our work within a broader context. We found that using the illustration as a vehicle to involve the community group members in the analysis and interpreting of the findings was a particularly engaging approach, addressing some of the language barriers to meaningful involvement in health research that would have been present if we had shared the written checklist.. However, in the process of producing and refining the illustration, we experienced challenges in developing a resource with text (albeit minimal text) that was truly accessible, as people may speak different dialects under the umbrella of one language, and people may speak a particular language but not read it.

In reflecting on the limitations, we acknowledge that the scope of this work was limited. Our focus was on understanding how we can improve on the involvement of community groups in health research, and we did not address research participation in our work. Diversity has many dimensions, and our checklist was developed through researchers working with three community groups based in the United Kingdom comprising specific ethnicities. It is important to note that although we focus here on the ethnicity of the community groups, members are likely to be at the intersection of multiple factors of marginalisation, as described by the NIHR [23]; for example language, being at extremes of age (over 75), being digitally excluded, being carers, having physical disabilities and multiple health conditions. There are many underserved communities who may have different individual considerations and contexts, and their knowledge and experience were not captured in our guidance. However, there are key areas of synergy with international guidance on involving communities in health research, such as Indigenous communities in Australia, New Zealand, Canada, and the USA, including the importance of building trust [24, 25]. However, we view our work as a launch pad for others to add to, with the aim of developing and sharing a growing body of knowledge to support and promote more diverse involvement of people in health research that is relevant to specific community groups. We also acknowledge that work in this area is still in its infancy, and while development of guidance makes a positive contribution, further work is needed to understand how best to implement the guidance, identify areas for refinement and evaluate impact.

## Conclusion

The development of this practical guidance on how to approach PCIE in an inclusive, acceptable and appropriate way can support researchers and contribute towards addressing equity in health research. Building positive and two-way relationships between researchers and community groups is an essential first step to inclusive involvement of communities in health research. To foster and maintain such relationships requires time. However, it is often difficult to gain funding for these activities that underpin good community-based involvement in health research, because they do not lead to an objective, measurable ‘output’. In the UK, there are now some funding schemes that support focused work to develop new relationships and partnerships with non-academic communities and organisations, however maintaining these relationships in the longer-term is challenging given the current approach of funders to fund project-specific PCIE activities rather than support longer-term community relationships. A shift in research culture and funding approach is needed to support meaningful and sustainable involvement of communities as partners in health research.

### Supplementary Information

**Additional file 1**. GRIPP-2 short-form.

## Data Availability

Data sharing is not applicable to this article as no datasets were generated or analysed during the current study.
