# Supplementary material for: Inclusive approaches to involvement of community groups in health research: the co-produced CHICO guidance
Source: Res Involv Engagem. 2023 Sep 7;9:76. doi: 10.1186/s40900-023-00492-9 (PMC10486022; doi:10.1186/s40900-023-00492-9)
Supplement: Supplementary file 1 — Additional file 1. GRIPP-2 short-form. [file 40900_2023_492_MOESM1_ESM.docx]

**GRIPP-2 Short Form**

| **Section and topic** | **Item** |
| --- | --- |
| 1: Aim  Report the aim of the study | To develop guidance on good practice for inclusive community involvement in health research through co-working with a South Asian, African Caribbean and (majority) Somali community group in Bristol. |
| 2: Methods  Provide a clear description of the methods used for patient and public involvement (PPI) in the study | We worked closely with community group leaders to understand the communities and build relationships before holding a series of meetings with each group. We worked with an illustrator to create a visual output, as suggested by group leaders. We used informal and open discussions tailored in format to each group, based on how each group works best. We carried out at least three meetings with each group to cover fair and inclusive ways of working, editing of terms and images and discussion on where to share the illustration. We paid for community leaders’ time, interpreters and transport where needed, as well as contributing towards activities and catering as suggested by group leaders. |
| 3: Results  Outcomes—Report the results of PPI in the study, including both positive and negative outcomes | We have created an illustration to share the key points for fair and inclusive working with these communities. We have also distilled the discussion points into a tabulated checklist for researchers to use to guide future work with community groups. Through this work, we have established trusted and reciprocal relationships with the three groups and we continue to work together. We are now discussing the research priorities of the groups to take forward to researchers, as described as the first step on the INCLUDE Roadmap. |
| 4: Discussion  Outcomes—Comment on the extent to which PPI influenced the study overall. Describe positive and negative effects | Involvement was at the core of this work. Discussion with group leaders suggested that a visual output with few words would work best for dissemination, to aid with comprehension where language and dialect may be a barrier. Where appropriate, in future we plan to work with community members to translate the text into the top languages spoken by each group (Urdu, Punjabi, Somali, Arabic). We worked with members of the community groups to understand barriers to involvement. We tabulated the results and compared group responses, which, with the exception of translation and separate gender groups (not required for Malcolm X) the same common themes appeared amongst the groups. |
| 5: Reflections  Critical perspective—Comment critically on the study, reflecting on the things that went well and those that did not, so others can learn from this experience | We have established healthy and reciprocal relationships with the three groups worked with. These relationships continue.  We hope that some of the items on the checklist can be used to form good working relationships with further community groups.  We have received feedback from one community leader that since this project, they have felt much more able to request appropriate financial input and refer other researchers to our illustration. Requests from researchers have historically taken up a lot of the leader’s time and we feel happy that this co-produced tool is of use to them.  We are aware that there are many marginalised groups in healthcare and healthcare research and that we have worked with three groups based upon their ethnicity. We see this work as part of a larger movement to ensure equitable representation in healthcare research.  While this work highlights the need for ongoing relationships, we recognise that research funding is often restricted to the lifetime of specific grants. This can further exacerbate the sometimes fragile relationships between research teams and communities. We would recommend the longer term, core funded employment of a coordinator or link worker to enable relationships to be maintained long-term.  We recognise that despite one of the community groups comprising multiple spoken languages, we have produced the illustration with English captions. We plan to gain funding in future to translate into the top languages identified by that community, working with the community leaders/members of the community to ensure culturally appropriate interpretation. |
